# Neuroprotective effect of masitinib in rats with postischemic stroke

**DOI:** 10.1007/s00210-014-1061-6

**Published:** 2014-10-26

**Authors:** Ivan Kocic, Przemyslaw Kowianski, Izabela Rusiecka, Grazyna Lietzau, Colin Mansfield, Alain Moussy, Olivier Hermine, Patrice Dubreuil

**Affiliations:** 1 Department of Pharmacology, Medical University of Gdansk, Debowa 23, 80-204 Gdansk, Poland; 2 Department of Anatomy and Neurobiology, Medical University of Gdansk, Gdansk, Poland; 3 Department of Clinical Sciences, Institute of Health Sciences, Pomeranian University of Slupsk, Slupsk, Poland; 4 AB Science, SA, Paris, France; 5 Université Paris Descartes, Service d’hématologie adulte, Centre de référence des mastocytoses, CNRS UMR 8147, Hôpital Necker, Paris, France; 6 Inserm U1068, Centre de Recherche en Cancérologie de Marseille, Signalisation, Hematopoiesis and Mechanisms of Oncogenesis, Centre de référence des mastocytoses; Institut Paoli Calmettes, CNRS, Aix Marseille Université, Marseille, France

**Keywords:** Masitinib, Tyrosine kinase inhibitor, Brain ischemic stroke, rt-PA, Brain ischemia

## Abstract

This study evaluated the therapeutic potential of masitinib, an oral tyrosine kinase inhibitor with activity against c-Kit and platelet-derived growth factor receptors (PDGFR), to reduce ischemic brain area and neurological deficit. Using a well-established filament model of ischemic stroke in rats, the responses to oral treatment with masitinib alone or in combination with recombinant tissue plasminogen activator (rt-PA) were compared to those after rt-PA (10 mg/kg intravenously (i.v.)) monotherapy. In both cases, two doses of masitinib were used—25 or 100 mg/kg, twice per day. Ischemic brain area and the neurological deficit were assessed using the triphenyltetrazolium chloride (TTC) method and behavioral neurological tests, respectively. Masitinib, as a single agent, reduced significantly the infarct size, as compared with the stroke control group. Brain ischemic area decreased from 9.14 to 4.36 % (25 mg/kg) or 2.60 % (100 mg/kg). Moreover, a combined treatment of masitinib with rt-PA produced a stronger effect than the one observed after each of the compound alone. The size of the brain ischemic area (rt-PA 1.67 %) was further reduced to 0.83 or 0.7 % at masitinib doses of 25 and 100 mg/kg, respectively. Masitinib reduced significantly brain ischemia induced by experimental stroke and potentiated the therapeutic effect of rt-PA.

## Introduction

Despite stroke being the second leading cause of mortality worldwide (Murray and Lopez 1977) and the third one of premature mortality in developed countries (MacDonald et al. 2000), it remains largely without any significant therapeutic strategies. Both ischemic and hemorrhagic strokes continue to have very poor outcomes (Lukovits and Goddeau 2011). Evidence-based strategies for treatment of ischemic stroke involve lowering blood pressure, reduction of cerebral edema, temperature control, glucose regulation, and antiplatelet and fibrinolytic treatments (Juvela et al. 1989; Kasner et al. 2002; Diringer and Zazulia 2004; Bereczki et al. 2000; McCormick et al. 2010). Recently, a new approach based upon neuroprotection has been proposed for the treatment of experimentally induced ischemic stroke in mice and rats (Rehni et al. 2011; Yuen et al. 2011).

Masitinib is an oral tyrosine kinase inhibitor that potently targets a limited number of kinases including c-Kit and platelet-derived growth factor receptors (PDGFR-α and PDGFR-β). Both kinases are implicated in diseases with inflammatory pathogenesis and various cancers (Dubreuil et al. 2009). Stem cell factor (SCF), the ligand of the c-Kit receptor, is a critical growth factor for mast cells. Masitinib can also regulate the activation of mast cells through its targeting Lyn and Fyn (Dubreuil et al. 2009). On the other hand, mast cells are known for their important role in sustaining the inflammatory network as they are one of the main central nervous system (CNS) sources of cytokines and chemokines (Stassen et al. 2002; Kinet 2007). Furthermore, perivascular localized mast cells secrete numerous vasoactive molecules and regulate blood-brain barrier (BBB) permeability (Esposito et al. 2002; Zhuang et al. 1996).

Additionally, according to several papers ( Lindsberg et al. 2010; Strbian et al. 2006; Strbian et al. 2007; Strbian et al. 2009), mast cells may actively participate in the pathogenesis of ischemic stroke.

Studies involving kinase inhibitor selectivity have shown that masitinib is one of the most selective kinase inhibitor under development (Anastassiadis et al. 2011). The drug’s selectivity effectively determines its activity and safety profile, i.e., potential for off-target effects and toxicity. Therefore, masitinib appears to be a good candidate for adjuvant use with current stroke management.

This study evaluated the therapeutic potential of masitinib to reduce infarct size and neurological deficit induced by a well-established intraluminal filament technique of ischemic stroke in animals (Chu et al. 2008). On this model, the responses of low and high doses of the drug given alone or in combination with recombinant tissue plasminogen activator (rt-PA) were investigated and compared to standard thrombolytic treatment (rt-PA).

## Materials and methods

### Study design and experimental animals

All experiments were performed on Wistar male rats, weighing between 270 and 350 g. Older rats were used because younger animals are more resistant to cerebral ischemia (Zhang et al. 2005). They were kept in a 12-h day and night cycle at room temperature, with access to water and food ad libitum. Body temperature and weight were recorded on a regular daily schedule. All procedures were carried out according to guidelines outlined by the European Community Council Directive of November 24, 1986 (86/609/EEC) and by the local Ethical Committee.

Masitinib, provided by AB Science (Paris, France), was administered orally as a suspension in distilled water by stomach plumb, twice per day at either 25 or 100 mg/kg body weight (b.w.). The first dose was applied 2 h poststroke and every 12 h thereafter for 7 days. The thrombolytic agent, rt-PA, was purchased from Boehringer Ingelheim, Germany. rt-PA was administered in slow intravenous (i.v.) infusion to the tail vein 2 h post stroke. All measurements and procedures took 7 days.

All experimental animals (*N* = 56) were divided into seven groups, with eight rats per group (Table 1). There were two control groups: I—a basic control group (BCG)—without stroke and treatment and II—a stroke control group (SCG)—with stroke and treatment with saline solution. The rest were five active treatment cohorts (III–VII), which comprised groups treated with rt-PA (10 mg/kg b.w.), masitinib (25 or 100 mg/kg b.w.), and rt-PA + masitinib (25 or 100 mg/kg b.w.).

**Table 1:** Experimental groups and procedures

Experimental group	Procedure
Basic control group, BCG	No stroke, no treatment
Stroke control group, SCG	Stroke with 0.9 % NaCl 2 h poststroke
rt-PA monotherapy	Stroke with rt-PA (10 mg/kg b.w.)
Masitinib (25 mg/kg) monotherapy	Stroke with masitinib only (25 mg/kg b.w. twice per day)
Masitinib (25 mg/kg) rt-PA combination	Stroke with rt-PA (10 mg/kg b.w.) plus masitinib (25 mg/kg b.w. twice per day)
Masitinib (100 mg/kg) monotherapy	Stroke with masitinib only at 100 mg/kg b.w. (twice per day)
Masitinib (100 mg/kg) rt-PA combination	Stroke with rt-PA (10 mg/kg b.w.) plus masitinib (100 mg/kg b.w. twice per day)

*b.w*. body weight, *rt*-*PA* recombinant tissue plasminogen activator

### Induction of stroke

Induction of anesthesia was conducted in an anesthetic chamber specifically adapted for small animal use by administration of 5.0 vol.% isoflurane (Abbot, USA) via Vaporizer (Rothacher, Swiss). Anesthesia was maintained during the surgical procedure with 3.8 vol.% isoflurane administered through a T-piece circuit that was directly connected to a close-fitting face mask. The animals were spontaneously ventilated with 50 vol.% oxygen mixed with ambient room air. During all procedures, the basic physiological parameters of experimental animals were monitored and maintained within physiological norms. Body temperature was kept at 36.5 to 37.5 °C. Permanent focal cerebral ischemia was experimentally induced by occlusion of the left middle cerebral artery (pMCAO) using the intraluminal filament technique (Longa et al. 1989). Briefly, a 4-0 monofilament nylon suture (Ethilon, UK), with heat-blunted tip, was inserted into the left external carotid artery and advanced through the internal carotid artery (at a depth of 15–17 mm) to occlude the origin of the middle cerebral artery. However, this method was modified by shortening of the suture (final length 2 cm). This modification led to prolongation of rat’s survival and enabled pharmacological manipulation within the period of 7 days (Lin et al. 2006). Following surgery, the animals were allowed to recover spontaneous breathing and were kept under standard conditions with free access to food and water.

### Behavioral tests

Animals were qualified for further study based on their extent of neurological deficit. This was assessed using the Bederson scale, 60 min after surgery (to determine success of the occlusion), again 48 h post stroke, and finally immediately prior to sacrifice (Bederson et al. 1986a, b; Schaar et al. 2010). The test was performed at approximately the same time each morning and recorded by a video camera. Rats were scored according to the following scale: 0 = no deficit; 1 = failure to extend right forepaw; 2 = decreased resistance to lateral push or decreased grip of right forelimb while tail pulled; and 3 = spontaneous circling or walking to the contralateral side.

Two other behavioral tests, the cylinder test (assessing spontaneous forelimb use) and the corner test (assessing sensorimotor and postural asymmetries) were performed at time-points of day 2 and 7 after stroke induction. In the cylinder test, a rat is observed in a transparent Plexiglas cylinder (20 cm diameter, 30 cm height). The number of independent wall placements observed for the right forelimb, left forelimb, and both forelimbs simultaneously were recorded by a video camera during 5 min (Schallert and Woodlee 2005).

In the corner test, the rat was placed in a cage with one side angled at 30° and recorded for 20 min. Normal subjects do not exhibit a turning preference, but after ischemia, test subjects have a turning preference to the non-impaired side. After the rat approached the corner, it was returned to the starting position. The number of left or right corner choices was recorded and expressed as a percentage of all attempts (Schallert and Woodlee 2005).

### Physiological parameters

Physiological parameters as degree of consciousness is presented according to the listed scale below: 1 = normal; 2 = mildly reduced; 3 = severely reduced; and 4 = comatose or dead.

### 2,3,5-Triphenyltetrazolium chloride staining

The rats were sacrificed 7 days after pMCAO, their brains immediately removed from the cranium and cooled to 4 °C. Five, 2-mm-thick coronal sections were made starting from the frontal pole to the occipital one. The brain slices were placed in 1 % solution of 2,3,5-triphenyltetrazolium chloride (TTC) (Sigma-Aldrich, Germany) in phosphate buffered saline (PBS, pH 7.4) and incubated in a dark humid chamber (37 °C) for 30 min. Gentle stirring and reversing of the plates ensured exposure of the slices to uniform staining. The sections were then washed three times with PBS and analyzed. In the intact brain, TTC is reduced by succinate dehydrogenase to its red insoluble form—formazan, while ischemic brain areas with inactive enzymes are not stained and appear pale (Bederson et al. 1986a, b; Joshi et al. 2004). Subsequently, each slice was photographed and the area of infarction, as well as the whole brain section border, was outlined using an image-analyzing program (AxioVision 4.8, Zeiss, Germany).

### Statistics

The extent of brain damage was calculated as follows: the area of ischemia for a given rat was expressed as a percentage of the total surface area of every individual rat brain, taken as 100 %. Each brain was cut into five equal sections. In each section, the damaged area was measured and expressed as a percentage of the total section area. Next, the mean value of the ischemic area for the whole group (eight animals) was calculated and compared with those obtained for the remaining experimental groups (calculated using the same method).

To calculate statistical significance of differences in ischemic area between experimental groups, the surface area was additionally expressed in number of pixels. Two-way ANOVA (Origin 8.5 and STATISTICA version 9.1) was used for calculation of statistical significance. A difference between results with *p* values <0.05 was considered to be statistically significant.

## Results

### Infarct size evaluation with TTC staining in rats with stroke and after treatment

**Fig. 1:**
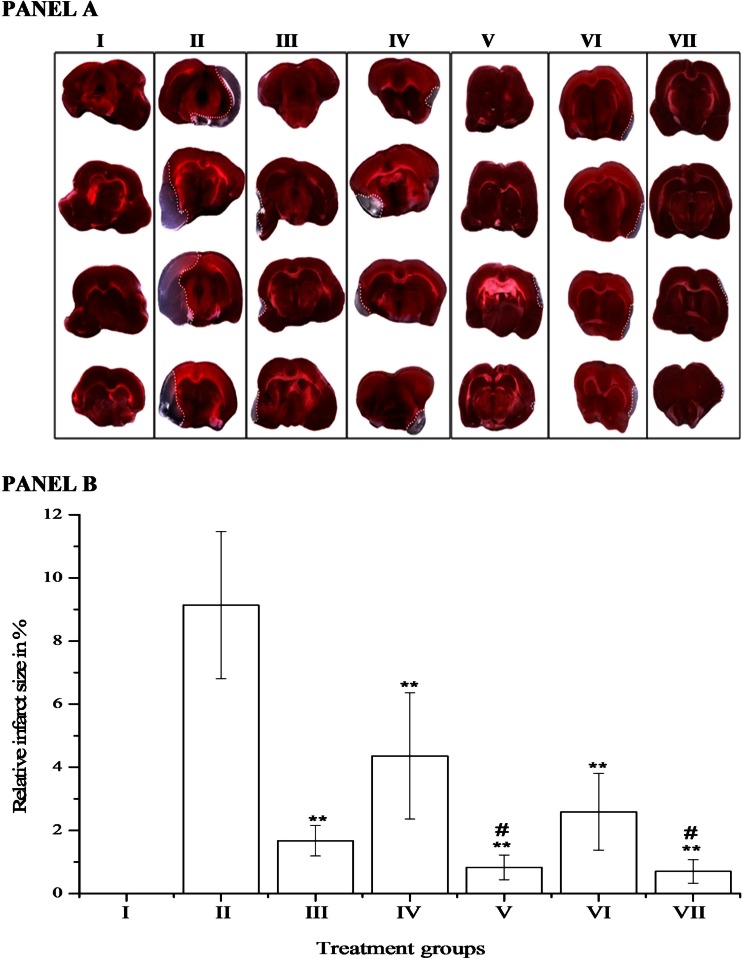
TTC images of brain sections and relative infarct size in experimental groups. **a** and **b** *I*—basic control group (BCG); *II*—stroke control group (SCG); *III*—rt-PA monotherapy; *IV*—masitinib (25 mg/kg) monotherapy; *V*—masitinib (25 mg/kg) + rt-PA; *VI*—masitinib (100 mg/kg) monotherapy; and *VII*—masitinib (100 mg/kg) + rt-PA. All data in (**b**) represent the mean ± S.E.M. **p < 0.01, statistically significant, as compared to stroke control group (SCG); #*p* < 0.05, statistically significant, as compared to rt-PA monotherapy group

The results obtained from all experimental groups are collected in Fig. 1 (Fig. 1a, b).

Representative images of coronal sections of each group are shown in subpanel a. The selection of the section side was determined by the distinctness of the ischemic area. Subpanel b presents relative ischemic brain areas for the treatment groups (calculated on the basis of coronal sections).

Figure 1a column I presents brain structure and morphology in the group of rats without stroke and any treatment (BCG), whereas column II presents poststroke brains with visible areas of ischemia (SCG) and clearly distinguished edges between infracted and normal tissues. These changes are further shown in a graphical presentation (subpanel b, bars I and II). An average brain ischemic area of SCG exceeds 9 %. Next images and bars (III–VII) of subpanels a and b, respectively, represent data of treatment groups. Number III of the image and bar reflects changes after standard fibrinolytic therapy— rt-PA. The ischemic area here is reduced up to 1.67 % and is statistically significant in comparison to that of SCG. Masitinib administered in the dose of 25 or 100 mg/kg b.w. reduced the infarct size to the values of 4.7 and 2.6 %, respectively (subpanel a images IV and VI; subpanel b bars IV and VI). In other words, there was a 49 % reduction in the case of the smaller dose and 72 % of the higher one. The most evident response is observed in the two groups treated with masitinib plus rt-PA (subpanel a, images V and VII; subpanel B bars V and VII). Indeed, this improvement of action (a reduction of brain ischemic area to 0.83 or 0.70 % for each of the dose of masitinib) was statistically significant (*p* < 0.05), as compared with rt-PA monotherapy.

Additionally, we have compared the volume and weight of the brains from control and masitinib-treated (with both doses) groups and found no statistically significant differences between them (data not shown).

### Behavioral observation

Generally, since the poststroke neurological deficit was rather small, its expected impact on animals’ behavior could also be only modest. This suggestion is confirmed by the results of the behavioral tests. Moreover, the results obtained after both doses of masitinib given alone or in combination with rt-PA are a little bit ambiguous.

As compared to SCG, masitinib given alone reduced neurological symptoms of stroke particularly if administered at a dose of 100 mg/kg. The effect of the drug became visible in the Benderson test after 2 and 7 days of stroke induction. On the other hand, if combined with rt-PA, masitinib also in a dose of 25 mg/kg produced an improvement in neurobehavioral deficit which was mainly noticed in the corner (*p* < 0.05) and cylinder tests. Comparable results were obtained in all tests with a dose of 100 mg/kg of masitinib plus rt-PA after 2 and 7 days. However, the differences did not reach statistical significance (Table 2).

**Table 2:** The results of different kinds of behavioral tests expressed in the number of appropriate movement (further explanation in the text)

Kind of behavioral test	*T* [days] after inducing stroke, when test was performed	Legs	Group I	Group II	Group III	Group IV	Group V	Group VI	Group VII
Benderson	2		0 ± 0	0.125 ± 0.33	0 ± 0	0.625 ± 0.67	0 ± 0	0.375 ± 0.48	0.375 ± 0.48
	7		0 ± 0	0.125 ± 0.33	0 ± 0	0 ± 0	0 ± 0	0.25 ± 0.66	0 ± 0
Cylinder	2	L	7.625 ± 4.41	1.625 ± 1.22	1.375 ± 1.73	3.125 ± 2.93	2.375 ± 1.58	1.25 ± 1.3	0.5 ± 1
		R	6.125 ± 2.67	1.5 ± 1.12	2.25 ± 1.3	2.75 ± 1.2	3.125 ± 2.52	0.5 ± 0.5	1.375 ± 1.49
		B	1.25 ± 1.3	0.375 ± 0.7	0.25 ± 0.43	0.625 ± 0.86	0.625 ± 0.7	0 ± 0	0.625 ± 0.7
	7	L	3.375 ± 2.12	2.625 ± 2	2.25 ± 0.22	1.625 ± 1.11	0.75 ± 1.64	0.625 ± 1.11	0 ± 0
		R	5 ± 3.46	2.5 ± 2.65	4.75 ± 4.41	1 ± 1.12	0.625 ± 0.7	0.5 ± 0.71	0.125 ± 0.33
		B	0 ± 0	0.25 ± 0.43	0 ± 0	0.25 ± 0.43	0.125 ± 0.33	0 ± 0	0 ± 0
Corner	2	L	4.125 ± 1.16	0.75 ± 0.82	0.25 ± 0.43	0.5 ± 0.7	1.125 ± 1.05	1 ± 1.32	0.25 ± 0.43
		R	5.875 ± 1.16	0.375 ± 0.48	1 ± 0.5	0.875 ± 1.36	1 ± 1	1 ± 0.86	0.125 ± 0.33
	7	L	4.625 ± 0.99	1.875 ± 2.52	0.25 ± 0.43	1.375 ± 1.99	1.5 ± 1.58	0.5 ± 0.7	0 ± 0
		R	4.875 ± 1.05	1.625 ± 2.17	0.375 ± 0.69	1.75 ± 1.29	0.875 ± 0.92	0.25 ± 0.43	0.375 ± 0.48

The values are means ± SEM calculated for each experimental group (*n* = 8 for every group, no significant differences were detected)

*T* number of poststroke days after which the test was performed, *L* left leg, *R* right leg, *B* both legs

### Physiological parameters

Masitinib induced sedation in all treated groups. The degree of sedation was dose-dependent and became noticeable when given alone or in combination with rt-PA. At the smaller dose (25 mg/kg), masitinib produced mild sedation (second level of the consciousness scale) and at the higher dose (100 mg/kg) a severe sedation (third level of the consciousness scale).

A weight loss (ca. 30 g) was observed in all treatment groups. Additionally, rats treated with masitinib (100 mg/kg) plus rt-PA suffered from permanent diarrhea.

## Discussion

Rat models of stroke are highly rated as inexpensive, reproducible, and valuable because of significant similarity between human and rat brain circulation (MacLellan et al. 2010). Additionally, this species of rodents characterizes a natural ability to develop cerebral collateral circulation leading to increase in angiogenesis in the postischemic region of the brain (Lin et al. 2008). The experiments presented in this study were performed on a model of stroke using an intraluminal filament technique, in which occlusion of the middle cerebral artery led to stroke with an average cerebral ischemic area of about 9 % and mortality rate less than 10 % during 7 days. This model provides an important advantage over the embolic model of stroke in terms of mortality rate, which by comparison is approximately 80 % during 2 days with a corresponding brain ischemic area of more than 50 % (Overgaard et al. 1992). Although neurological deficit was not so expressive in the model used in this study, it permitted evaluation of the effects of pharmacological intervention on brain infarct size which would not be possible with an embolic model of cerebral ischemia.

The results presented here showed that masitinib reduced stroke-related brain infarct size on the model of stroke used in this study. The most prominent neuroprotective effects of the drug (evidenced by a reduced infarct size) became visible if it was administered together with rt-PA. Moreover, these responses reached statistical significance, as compared to the treatment with rt-PA alone (SCG). Thus, the results mentioned above may suggest that masitinib given together with rt-PA produced an additive therapeutic effect on poststroke rats. To elucidate this additive action of both compounds, it should be first carried out an analysis of the responses produced by each of the single agent, i.e., by rt-PA or masitinib given alone.

Thrombolysis using rt-PA is the standard treatment for acute ischemic stroke. However, there have been conflicting data obtained concerning the effects of rt-PA after focal brain ischemia. Even an aggravation of the injury has been noticed, but mainly if rt-PA was used in mice (Wang et al. 1998). However, the results of the experiments presented here, which were carried out on rats, confirmed the therapeutic effect of this drug. In relation to the existing data, the clinical problem at hand is how to increase the overall efficacy of rt-PA and to expand its therapeutic window, particularly by reducing or eliminating the risk of hemorrhagic transformation (del Zoppo and Hallenbeck 2000; Wang et al. 2004) which increases with delayed administration of this agent.

In the light of the available research, it is difficult to put forward a definite explanation of the neuroprotective effect of masitinib. The therapeutic effect of the drug in question was smaller than that produced by rt-PA. The latter observation seems not to be surprising as there are several studies in which usage of neuroprotectants without thrombolysis derived reperfusion was not very effective (Liu et al. 2010).

One of the possible mechanisms involved in this neuroprotection may be related to masitinib’s well-known inhibitory action on tyrosine kinases, especially c-Kit and platelet-derived growth factor receptors (e.g., PDGFR-β). The latter seems to be important in the light of recent finding that stimulation of PDGFR-β can disintegrate BBB in early phase of brain ischemia (Shen et al. 2012). It has been shown that both kinases are implicated in inflammatory diseases, as well as various cancers, and PDGFR signaling regulates cerebrovascular permeability after stroke (Su et al. 2008). Thus, it may be assumed that inhibition of PDGFR participated in the neuroprotection of masitinib which was noticed in this study. Similarly, a therapeutic potential of this drug has been demonstrated in humans via proof-of-concept studies in such neurological illnesses as Alzheimer’s disease (Piette et al. 2001) and multiple sclerosis (Vermersch et al. 2012).

Another mechanism which could explain the neuroprotective effect of masitinib is inhibition of mast cell activity in the CNS or outside the brain. Different teams of researchers found an increased presence of mast cells and their rapid degranulation in association with morphologic changes of ischemic brain injury (Lindsberg et al. 2010; Strbian et al. 2006). This phenomenon led further to an augmentation of postischemic BBB leakage and also contributed to hemorrhagic transformation (Tejima et al. 2007; Lindsberg et al. 2010). Additionally, confirmation of mast cell contribution to stroke was found on the transient MCAO model in rats in which pharmacological mast cell stabilization with sodium cromoglycate reduced the deleterious symptoms of stroke (Strbian et al. 2009).

With respect to masitinib, the present study showed that this drug reduced significantly (<0.05) the infarct territory in a dose-dependent manner. This effect appeared despite its poor BBB penetration. On the other hand, possible BBB leakage, a characteristic feature of stroke condition, usually provides a therapeutic opportunity for drugs that normally cannot cross the BBB (Lin et al. 2006). However, lack of significant differences in volume and weight of control and masitinib-treated brains after the stroke in our experimental model suggests negligible participation of this scenario (data not shown).

Although we cannot provide further experimental data for masitinib regarding direct brain effects related to neuronal survival, affecting BBB or inflammation in stroke, we have putative evidence from scientific literature that tyrosine kinase inhibitors (including masitinib) poorly cross BBB, and even if they can cross in a small amount they are quickly eliminated from brain tissue by P-glycoprotein (ABCB1 and ABCG2) (Lagas et al. 2009). Masitinib is known as a strong inhibitor of PDGFR, but extracerebral inhibition of mast cell recruitment is probably the most important component of preserving BBB integrity and has highly significant impact on neuroinflammatory cascade and activity of microglia in the brain tissue (Skaper et al., 2012; Skaper et al. 2014).

The brief considerations concerning concepts of both rt-PA and masitinib actions permit a better understanding and thereby explanation of the additive interaction which was observed here in the presented paper. The augmentation of rt-PA action by masitinib may result from the fact that such combination therapy targets a different pathway of ischemic cascade, activated during stroke, and masitinib preserves BBB against disintegration in the presence of rt-PA. Similar suppositions for explaining neuroprotective action of other agents in stroke were presented by Strbian et al. (2009) or Liu et al. (2010).

Theoretically, the size of brain ischemic area should be expressed by behavioral changes in poststroke animals. However, such compatibility was not always observed in the behavioral tests performed in this study. Perhaps, this incompatibility results from the limitations of the stroke model itself. As it has already been mentioned, the size of ischemia here was smaller than that in the classical pMCAO model. Thus, the present results obtained from behavioral tests showed no more than a trend towards reduced neurological symptoms of stroke in rats receiving masitinib with or without rt-PA. Statistically significant values were only observed if masitinib (25 mg/kg) plus rt-PA was investigated in the corner test.

With respect to adverse drug reactions, masitinib produced sedation which was especially visible after the dose of 100 mg/kg. Additionally, rats treated with this dose suffered from permanent diarrhea.

To conclude, this study clearly demonstrated that masitinib represents an attractive potential therapy of ischemic stroke. Particularly promising seems to be the dose of 25 mg/kg, as it decreased ischemic area by approximately 50 or 90 %, if the drug was used alone or in combination with rt-PA, respectively. These therapeutic actions were accompanied by manageable side effects. Further increase in the dose of masitinib appears to be irrational because it led to more severe side effects with lack of improvement in therapeutic action.

Summarizing, masitinib may be recommended as an appropriate candidate for a neuroprotective strategy, especially in the acute stage of stroke, the aim of which is to improve the final outcome of the therapeutic intervention of ischemic stroke with rt-PA and preserve BBB integrity. Therefore, the drug deserves further clinical investigation and further research to corroborate this study’s experimental observations and better understand masitinib’s mechanisms of action in stroke.

## Abbreviations

BCG: Basic control group
b.w.: Body weight
BBB: Blood-brain barrier
i.v.: Intravenous
CNS: Central nervous system
pMCAO: Permanent middle cerebral artery occlusion
rt-PA: Recombinant tissue plasminogen activator
SCG: Stroke control group
TTC: Triphenyltetrazolium chloride

## References

[CR1] Anastassiadis T, Deacon SW, Devarajan K, Ma H, Peterson JR (2011). Comprehensive assay of kinase catalytic activity reveals features of kinase inhibitor selectivity. Nat Biotechnol.

[CR2] Bederson JB, Pitts LH, Tsuji M, Nishimura MC, Davis RL, Bartkowski H (1986). Rat middle cerebral artery occlusion: evaluation of the model and development of a neurologic examination. Stroke.

[CR3] Bederson JB, Pitts LH, Germano SM, Nishimura MC, Davis RL, Bartkowski HM (1986). Evaluation of 2,3,5-triphenyltetrazolium chloride as a stain for detection and quantification of experimental cerebral infarction in rats. Stroke.

[CR4] Bereczki D, Liu M, Prado GF, Fekete I (2000). Cochrane report: a systematic review of mannitol therapy for acute ischemic stroke and cerebral parenchymal hemorrhage. Stroke.

[CR5] Chu X, Qi C, Zou L, Fu X (2008). Intraluminal suture occlusion and ligation of the distal branch of internal carotid artery: an improved rat model of focal cerebral ischemia-reperfusion. J Neurosci Meth.

[CR6] del Zoppo GJ, Hallenbeck JM (2000). Advances in the vascular pathophysiology of ischemic stroke. Thromb Res.

[CR7] Diringer MN, Zazulia AR (2004). Osmotic therapy: facts and fiction. Neurocrit Care.

[CR8] Dubreuil P, Letard S, Ciufolini M, Gros L, Humbert M, Casteran N (2009). Masitinib (AB1010), a potent and selective tyrosine kinase inhibitor targeting KIT. PLoS One.

[CR9] Esposito P, Chandler N, Kandere K, Basu S, Jacobson S, Connolly R (2002). Corticotropin-releasing hormone and brain mast cells regulate blood–brain-barrier permeability induced by acute stress. J Pharmacol Exp Ther.

[CR10] Joshi CN, Jain SK, Murthy PS (2004). An optimized triphenyltetrazolium chloride method for identification of cerebral infarcts. Brain Res Protoc.

[CR11] Juvela S, Heiskanen O, Poranen A, Valtonen S, Kuurne T, Kaste M (1989). The treatment of spontaneous intracerebral hemorrhage. A prospective randomized trial of surgical and conservative treatment. J Neurosurg.

[CR12] Kasner SE, Wein T, Piriyawat P, Villar-Cordova CE, Chalela JA, Krieger DW (2002). Acetaminophen for altering body temperature in acute stroke: a randomized clinical trial. Stroke.

[CR13] Kinet JP (2007). The essential role of mast cells in orchestrating inflammation. Immunol Rev.

[CR14] Lagas SJ, van Waterschoot RAB, van Tilburg VACJ, Hillebrand MJ, Lankheet N, Rosing H (2009). Brain accumulation of dasatinib is restricted by P-glycoprotein (ABCB1) and breast cancer resistance protein (ABCG2) and cab be enhanced by elacridar treatment. Clin Cancer Res.

[CR15] Lin TN, Cheung WM, Wu JS, Chen JJ, Lin H, Chen JJ, Liou JY, Shyue SK, Wu KK (2006). 15d-prostaglandin J2 protects brain from ischemia-reperfusion injury. Arterioscler Thromb Vasc Biol.

[CR16] Lin CY, Chang C, Cheung WM, Lin MH, Chen JJ, Hsu CY, Chen JH, Lin TN (2008). Dynamic changes in vascular permeability, cerebral blood volume, vascular density, and size after transient focal cerebral ischemia in rats: evaluation with contrast-enhanced magnetic resonance imaging. J Cereb Blood Flow Metab.

[CR17] Lindsberg PJ, Strbian D, Karjalainen-Lindsberg ML (2010). Mast cells as early responders in the regulation of acute blood-brain barrier changes after cerebral ischemia and hemorrhage. J Cereb Blood Flow Metab.

[CR18] Liu R, Liu Q, He S, Simpkins JW, Yang SH (2010). Combination therapy of 17beta-estradiol and recombinant tissue plasminogen activator for experimental ischemic stroke. J Pharmacol Exp Ther.

[CR19] Longa EZ, Weinstein PR, Carlson S, Cummins R (1989). Reversible middle cerebral artery occlusion without craniectomy in rats. Stroke.

[CR20] Lukovits TG, Goddeau RP (2011). Critical care of patients with acute ischemic and hemorrhagic stroke: update on recent evidence and international guidelines. Chest.

[CR21] MacDonald BK, Cockerell OC, Sander JWAS, Shorvon SD (2000). The incidence and lifetime prevalence of neurological disorders in a prospective community-based study in the UK. Brain.

[CR22] MacLellan CL, Silasi G, Auriat AM, Colbourne F (2010). Rodent models of intracerebral hemorrhage. Stroke.

[CR23] McCormick M, Hadley D, McLean JR, Macfarlane JA, Condon B, Muir KW (2010). Randomized controlled trial of insulin for acute poststroke hyperglycemia. Ann Neurol.

[CR24] Murray CJL, Lopez AD (1977). Mortality by cause for eight regions of the world: global burden of disease study. Lancet.

[CR25] Overgaard K, Sereghy T, Boysen G, Pedersen H, Diemer NH (1992). Reduction of infarcts volume and mortality by thrombolysis in a rat embolic stroke model. Stroke.

[CR26] Piette F, Belmin J, Vincent H, Schmidt N, Pariel S, Verny M (2001). Masitinib as an adjunct therapy for mild-to-moderate Alzheimer’s disease: a randomized, placebo-controlled phase 2 trial. Alzheimer Res Ther.

[CR27] Rehni AK, Singh TG, Kakkar T, Arora S (2011). Involvement of src-kinase activation in ischemic preconditioning induced protection of mouse brain. Life Sci.

[CR28] Schaar KL, Brenneman MM, Savitz SI (2010). Functional assessments in the rodent stroke model. Exp Transl Stroke Med.

[CR29] Schallert T, Woodlee MT, Whishaw I, Kolb B (2005). A handbook with tests. Behavior of the laboratory rat.

[CR30] Shen J, Ishii Y, Xu G, Dang TC, Hamashima T, Matsushima T (2012). PDGFRβ as a positive regulator of tissue repair in a mouse model of focal cerebral ischemia. J Cereb Blood Flow Metab.

[CR31] Skaper SD, Giusti P, Facci L (2012). Microglia and mast cells: two tracks on the road to neuroinflammation. FASEB J.

[CR32] Skaper SD, Facci L, Guisti P (2014). Mast cells, glia and neuroinflammation: partners in crime?. Immunol.

[CR33] Stassen M, Hültner L, Müller C, Schmitt E (2002). Mast cells and inflammation. Arch Immunol Ther Exp.

[CR34] Strbian D, Karjalainen-Lindsberg M-L, Tatlisumak T, Lindsberg PJ (2006). Cerebral mast cells regulate early ischemic brain swelling and neutrophil accumulation. J Cereb Blood Flow Metab.

[CR35] Strbian D, Karjalainen-Lindsberg ML, Kovanen PT, Tatlisumak T, Lindsberg PJ (2007). Mast cell stabilization reduces hemorrhage formation and mortality after administration of thrombolytics in experimental ischemic stroke. Circ.

[CR36] Strbian D, Kovanen PT, Karjalainen-Lindsberg ML, Tatlisumak T, Lindsberg PJ (2009). An emerging role of mast cells in cerebral ischemia and hemorrhage. Ann Med.

[CR37] Su EJ, Fredriksson MG, Folestad E, Cale J, Andrae J, Gao Y (2008). Activation of PDGF-CC by tissue plasminogen activator impairs blood brain barrier integrity during ischemic stroke. Nat Med.

[CR38] Tejima E, Zhao BQ, Tsuji K, Rosell A, van Leyen K, Gonzalez RG (2007). Astrocytic induction of matrix metalloproteinase-9 and edema in brain hemorrhage. J Cereb Blood Flow Metab.

[CR39] Vermersch P, Benrabah R, Schmidt N, Zéphir H, Clavelou P, Vongsouthi C (2012). Masitinib treatment in patients with progressive multiple sclerosis: a randomized pilot study. BMC Neurol.

[CR40] Wang YF, Tsirka SE, Strickland S, Stieg PE, Soriano SG, Lipton SA (1998). Tissue plasminogen activator (tPA) increases neuronal damage after focal cerebral ischemia in wild-type and tPA-deficient mice. Nat Med.

[CR41] Wang X, Tsuji K, Lee SR, Ning M, Furie KL, Buchan AM (2004). Mechanisms of hemorrhagic transformation after tissue plasminogen activator reperfusion therapy for ischemic stroke. Stroke.

[CR42] Yuen CM, Sun CK, Lin YC, Chang LT, Kao YH, Yen CH (2011). Combination of cyclosporine and erythropoietin improves brain infarct size and neurological function in rats after ischemic stroke. J Transl Med.

[CR43] Zhang S, Boyd J, Delaney K, Murphy TH (2005). Rapid reversible changes in dendritic spine structure in vivo gated by the degree of ischemia. J Neurosci.

[CR44] Zhuang X, Silverman AJ, Silver R (1996). Brain mast cell degranulation regulates blood-brain barrier. J Neurobiol.

